# Complete Genome Sequence of *Escherichia coli* O157:H7 Bacteriophage phiJLA23 Isolated in Mexico

**DOI:** 10.1128/genomeA.00219-12

**Published:** 2013-02-28

**Authors:** Luis Amarillas, Cristobal Chaidez, Yadira Lugo, Josefina León-Félix

**Affiliations:** a Laboratorio de Microbiología Ambiental y de Alimentos, Centro de Investigación en Alimentación y Desarrollo A. C., Sinaloa, México; b Departamento de Biología Molecular del Instituto de Investigación Lightbourn A. C., Chihuahua, México; c Centro de Investigación y Asistencia en Tecnología y Diseño del Estado de Jalisco, Guadalajara, México

## Abstract

The bacteriophage phiJLA23 was isolated from an animal feces sample and lytic activity was demonstrated against the *Escherichia coli* O157:H7 strain. We report the complete nucleotide sequence of bacteriophage phiJLA23, information which may be useful for determining whether this phage is a candidate for biocontrol or another biotechnological application.

## GENOME ANNOUNCEMENT

*Escherichia coli* O157:H7 is an important human pathogen of worldwide importance that has been implicated in several outbreaks, and its antibiotic resistance is increasing (1, 2). The indiscriminate use of antibiotics results in the emergence of resistant bacterial strains (3), and the development of antibiotic resistance highlights the need for alternative strategies to combat pathogenic bacteria. Phages can be used as biocontrol agents against antibiotic-resistant bacteria (4).

We report the characterization of the complete genome sequence of the *E. coli* O157:H7 phage phiJLA23. This phage was isolated from a farm animal feces sample from the Culiacan Valley, Mexico, and showed high lytic activity on various strains of *E. coli* O157. To our knowledge, this is the first report of genomic sequencing of a coliphage isolated in Mexico. The phage DNA was isolated from purified phage particles by the phenol-chloroform method described by Sambrook and Russell (5) and was sequenced using the GS Junior 454 system platform (Roche Diagnostics, Mannheim, Germany) (6). Sequences were assembled and annotation data were processed using Geneious version 6.0.3 (7). Open reading frames (ORFs) were predicted using Glimmer 3.02 (8) and GeneMarkS version 4.7 (9).

The DNA sequence of phage phiJLA23 was determined, and bioinformatic analysis revealed that it consists of double-stranded DNA of 43,017 bp with an overall G+C content of 44.2%. A total of 65 ORFs were identified in the genome, and the translated ORF products were compared by using the Basic Local Alignment Search Tool (BLASTp) and an NCBI Conserved Domain Database search (10), which revealed only 62 ORFs that encode proteins with known functions. In general, functionally related ORFs are clustered in phage genomes.

Based on the predictions, this phage genome contains complete genes for phage structure and genes for replication; the genome encodes hypothetical proteins involved in the virion structure, assembly, and DNA replication of the bacteriophage. The replication gene module encodes replication and recombination proteins (DNA polymerase I, terminase large subunit, terminase small subunit, replication protein alpha, exodeoxyribonuclease, recombination protein, DNA binding protein, and ATP-dependent helicase). The phage structural genes encode minor capsid protein, major capsid protein, prohead protease, protein required for capsid morphogenesis, tail fiber protein, tail tape measure protein, and tail assembly protein. Furthermore, a putative endolysin gene was identified in the genome of the bacteriophage phiJLA23. According to the results of homology searching, the nucleotide sequence showed high similarity to the lysozyme of phage JK06.

The genome sequence of the bacteriophage phiJLA23 presents a high degree of similarity to the genome of phage JK06 (GenBank number DQ121662.1), with query coverage of 95% and an E value of 0.0. Bacteriophage JK06 is a specific type for the pathogenic *E. coli* O157:H7 (11).

### Nucleotide sequence accession number.

The complete genome sequence of bacteriophage phiJLA23 has been deposited in GenBank under accession number KC333879.
